# Nitroglycerin combined with NSAIDs for prevention of post-ERCP pancreatitis: A meta-analysis of prospective, randomized, controlled trials

**DOI:** 10.1097/MD.0000000000038764

**Published:** 2024-07-05

**Authors:** Xuan Zhang, Jing-Ming Zhang, Wan Wei, Hui Lin

**Affiliations:** a Fuzhou First General Hospital Affiliated with Fujian Medical University, China; b Department of Infectious and Hepatology Diseases, Fujian Medical University, Mengchao Hepatobiliary Hospital of Fujian Medical University; c The Affiliated Huai’an No. 1 People’s Hospital of Nanjing Medical University, Huaian, China; d Fuzhou First General Hospital Affiliated with Fujian Medical University, China.

**Keywords:** GTN, NSAIDs, PEP, post-ERCP pancreatitis

## Abstract

**Background:**

Acute pancreatitis is the most common complication of endoscopic retrograde cholangiopancreatography (ERCP), with an incidence of approximately 9.7% according to some literature reviews. Recent clinical guidelines propose that glyceryl trinitrate (GTN) can reduce the incidence of post-ERCP pancreatitis (PEP). However, currently, no guidelines provide an exact opinion on GTN and nonsteroidal anti-inflammatory drugs (NSAIDs) to prevent post-ERCP pancreatitis.

**Objective:**

A meta-analysis was performed of published, full-length, randomized controlled trials (RCTs) evaluating the effects of prophylactic use of GTN, including GTN alone or GTN in combination with NSAIDs, on the prevention of PEP.

**Methods:**

Literature searches were conducted using PubMed, Embase, Web of Science, and The Cochrane Library. Search terms included “endoscopic retrograde cholangiopancreatography” OR “ERCP,” “OR ‘PEP’ OR ‘post-endoscopic retrograde cholangiopancreatography pancreatitis’, pancreatitis,” “GTN” OR “glyceryl trinitrate” OR “nitroglycerin,” “NSAIDs” OR “Nonsteroidal Anti-inflammatory Drugs” and limited to RCT.

**Results:**

A total of 10 RCTs comprising 3240 patients undergoing ERCP were included. Meta-analysis revealed that the administration of GTN was associated with a significant reduction in the overall incidence of PEP. Moreover, PEP incidence was significantly lower in the GTN combined with the NSAIDs group than in the GTN alone group. GTN alone or GTN combined with NSAIDs may not reduce the severity of PEP (risk ratio = 0.64; 95% confidence interval: 0.41–0.99; *P* = .04). The difference in incidence between the 2 groups is 1.01% (6/594) in the GTN with NSAIDs group and 2.36% (14/592) in the placebo group.

**Conclusion:**

GTN has a significant benefit in preventing postoperative ERCP pancreatitis (*P* < .001). And neither GTN nor GTN plus NSAIDs reduces the incidence of non-mild ERCP postoperative pancreatitis. These conclusions need to be confirmed by high-quality randomized controlled studies with multicenter, large samples, and long-term follow-up.

## 1. Introduction

Since the advent of endoscopic retrograde cholangiopancreatography (ERCP) in the 20th century, its technological development has gradually matured. However, post-ERCP pancreatitis (PEP) is the most common complication with a high incidence and mortality rate. It has been the focus of research by digestive physicians. From the perspective of pharmacology, increased tonus of the sphincter of Oddi is a risk factor for the development of PEP; hence, drugs that relax smooth muscle cells have been proposed as prophylactic agents. Glyceryl trinitrate (GTN) is a NO (Nitric Oxido) donor with potent relaxant properties in smooth muscles, including sphincters. The 2019 European Society of Gastrointestinal Endoscopy (ESGE) updated the ERCP-related adverse event guidelines including the use of GTN before ERCP for the prevention of PEP in the recommended entry.^[1]^ ESGE also recommends routine rectal administration of diclofenac or indomethacin before ERCP, in patients with nonsteroidal anti-inflammatory drug (NSAID) contraindications. Hence, we conducted a comprehensive, updated meta-analysis that included a newly published randomized controlled trial (RCT) to examine the efficacy of prophylactic GTN combined with NSAID administration for PEP and whether it was effective in reducing the incidence of moderate-to-severe PEP.

## 2. Methods

### 2.1. Search criteria and research methods

Two researchers independently performed a comprehensive and systematic search of the literature up to September 2022 on PubMed, Embase, Web of Science, and The Cochrane Library. Search terms included “endoscopic retrograde cholangiopancreatography” OR “ERCP,” “post-ERCP pancreatitis” OR “PEP” OR “post-endoscopic retrograde cholangiopancreatography pancreatitis,” “pancreatitis,” “GTN” OR “glyceryl trinitrate” OR “nitroglycerin,” “NSAIDs” OR “Nonsteroidal Anti-inflammatory Drugs” and limited to RCTs. No language restriction was imposed. The search was limited initially to publications of RCTs. Furthermore, the recommendations and consensus on the complications of PEP by Cotton et al^[2]^ defined it as a new or worsened abdominal pain combined with >3 times the normal value of amylase or lipase at >24 hours requiring either hospital admission or prolongation of a planned admission. The severity classification of PEP was based on the recommendations of the updated *ERCP-related adverse events: ESGE Guideline*.^[3]^ The severity of acute pancreatitis according to the revised Atlanta classification includes mild acute pancreatitis, moderately severe acute pancreatitis, and severe acute pancreatitis. The specific standards of acute pancreatitis are reported in Table 1. A combination of pancreatitis other than mild acute pancreatitis is referred to as non-mild PEP.

**Table 1:** Atlanta Acute Pancreatitis Grading Scale.

Grades of severity
Mild acute pancreatitis
• No organ failure
• No local or systemic complications
Moderately severe acute pancreatitis
• Organ failure that resolves within 48 h (transient organ failure) and/or
• Local or systemic complications without persistent organ failure
Severe acute pancreatitis
• Persistent organ failure (>48 h)
- Single organ failure
- Multiple organ failure

The following selection criteria were applied: study design: prospective RCT of GTN in the prevention of post-ERCP pancreatitis versus placebo/blank; study population: patients undergoing ERCP; intervention: prophylactic administration of GTN alone, or prophylactic administration of GTN combination with; comparison intervention: placebo or no treatment; and outcome measures: the overall incidence of PEP, the incidence of moderate-to-severe PEP. Data collection and literature evaluation were performed by 2 reviewers independently. Review Manager 5.4 was used for statistical analysis.

### 2.2. Material inclusion and exclusion criteria

The following selection criteria were applied: study design: prospective RCT of GTN in the prevention of post-ERCP pancreatitis versus placebo/blank; study population: patients undergoing ERCP; intervention: prophylactic administration of GTN alone, or prophylactic administration of GTN combination with; comparison intervention: placebo or no treatment; and outcome measures: the overall incidence of PEP, the incidence of moderate-to-severe PEP. Data collection and literature evaluation were performed by 2 reviewers independently. Any of the following conditions were excluded in the study: other measures to prevent PEP other than nitroglycerin were used; the study is limited to the trial of drugs for the prevention and treatment of hyperamylamia or other complications other than pancreatitis after ERCP; the test has not been completed or only has preliminary results; the full text of the test report cannot be obtained.

### 2.3. Data extraction and study quality

Data from eligible studies were extracted independently by 2 reviewers using standard forms. Details of the studies including first author, year of publication, sample size, interventions, Control, dosage, follow-up, routes of drug administration, inclusion and exclusion criteria of each study, outcome (including the occurrence of PEP and the severity of PEP), adverse drug reactions (Table 2). Any disagreements were resolved by discussion and consensus. The quality of included trials was scored with the Jadad Scale^[14]^ which assesses descriptions of randomization, double-blinding, withdraws, or dropouts. The Jadad Scale ranges from 0 to 5 points, with a low-quality study of a score of less than 2 (including 2) and a high quality of a score of at least 3. Any disagreement was resolved by discussion between the 2 reviewers. The scores are shown in Table 3. The methodological quality was independently evaluated by 2 of the authors. Trials with a low risk of bias were those that adequately met the 3 criteria of generation of allocation sequence, allocation concealment, and double blinding. Trials with a moderate risk of bias were those where one or more of these 3 criteria were only partly met, while trials were considered to carry a high risk of bias if one or more criteria were not met. Any disagreement was resolved by discussion between the 2 reviewers. All analyses were based on previously published studies, thus no ethical approval and patient consent are required.

**Table 2:** Principal characteristics of the randomized studies.

Author, year	Study design	Patient characteristic		Intervention	Control	Medication route	Dosage	Sample size (I/C)	Outcome
		Mean age (I/C)	Female gender (I/C)						
Sudhindran et al 2001^[4]^	RCT	63.7/63.7	68/60	GTN	Placebo	Sublingual	2 mg	90/60	PEP, the severity of pancreatitis
Kaffes et al 2006^[5]^	RCT	60/65	62/65	GTN	Placebo	Patch	5 mg	155/163	PEP, the severity of pancreatitis
Moreto et al 2003^[6]^	RCT	66.7/65.2	27/30	GTN	Placebo	Patch	15 mg	71/73	PEP, the severity of pancreatitis
Beauchant et al 2008^[7]^	RCT	50/54	76/74	GTN	Placebo	Intravenous	0.1 mg + 35 µg/kg for 6 h	105/103	PEP, the severity of pancreatitis
Nojgaard et al 2009^[8]^	RCT	67/65	237/237	GTN	Placebo	Patch	15 mg	401/405	PEP, the severity of pancreatitis
Bhatia et al 2009^[9]^	RCT	42/42.5	88/79	GTN	No intervention	Patch	10 mg	124/126	PEP, the severity of pancreatitis
Hao et al 2009^[10]^	RCT	64.29 ± 13.40/63.36 ± 15.13	23/20	GTN	Placebo	Sublingual	5 mg	38/36	PEP
Tomoda et al 2019^[11]^	RCT	68/68	158/156	GTN + diclofenac	Diclofenac	Sublingual	5 mg	444/442	PEP, the severity of pancreatitis
Sotoudehmanesh et al 2014^[12]^	RCT	58.4 ± 17.8/58.6 ± 17.5	74/80	GTN + indomethacin	Placebo + indomethacin	Sublingual	5 mg	150/150	PEP, the severity of pancreatitis
Bo Luo & Wei 2016^[13]^	RCT	52.74 ± 5.40/54.30 ± 6.27	18/16	GTN + indomethacin	Indomethacin	Sublingual	0.5 mg	35/35	PEP

GTN = glyceryl trinitrate, RCT = randomized controlled trial, PEP = post-endoscopic retrograde cholangiopancreatography pancreatitis, I/C = Intervention/Control group.

**Table 3:** Characteristics of age and gender of randomized controlled trials.

Group	Age (y)	Female
Control	62.43 ± 10.20	831
Treatment	62.20 ± 10.08	817
χ²		0.017
*P*	.927	.895

### 2.4. Statistical analysis

All statistical analyses were performed using Review Manager (Version 5.4, Cochrane Collaboration, Oxford, UK). All outcomes were expressed as risk ratio (RR) with 95% confidence interval (CI). Heterogeneity was assessed by visual inspection of a forest plot, the Cochran *Q* test, and the *I*^2^ statistic. Heterogeneity was considered significant by the Cochran *Q* test with *P* < .05 or by *I*^2^ > 50%. A fixed-effects model or random-effects model was used, depending on the absence or presence of heterogeneity. We restrict the meta-analysis to the subgroups: use GTN alone; use GTN with NSAID. We also assessed the potential for publication bias shown as a funnel plot. A *P* value less than .05 was judged as statistically significant.

## 3. Results

### 3.1. Study selection and characteristics

The database search yielded 128 related articles, of which 87 were obtained after eliminating duplicates. After reading the titles and abstracts, the remaining 12 articles were initially screened, as they did not meet the requirements of a non-RCT. After reading the full text, based on the inclusion criteria, one document was excluded. One RCT was excluded because the control group was different. A total of 10 RCTs^[4–13]^ with 3240 patients undergoing ERCP were included in the study. Of the 10 RCTs, 7^[4–10]^ studies used GTN as the treatment group and placebo or no treatment as the control group; 3 studies^[11–13]^ were controlled by using GTN plus NSAIDs such as diclofenac and indomethacin, and diclofenac or indomethacin alone as the control group; all studies had clearly defined PEP, and all studies had clearly stated the dosage and route of the administration of the drug. Moreover, 9 studies^[4–9,11–13]^ documented the incidence of moderate-to-severe pancreatitis; 8 studies^[5–9,11–13]^ documented the occurrence of adverse effects.

### 3.2. Quality assessment

The 10 RCTs included in the study were published in full format. The participants had the same baseline age and sex. All included trials had clear inclusion criteria, with 8 trials^[4–6,8–11,13]^ establishing exclusion criteria, whereas 2 RCTs^[7,12]^ not describing the exclusion criteria. Successful randomization was completed in all trials, and all trial assessments were double-blind. Five trials^[4,5,8,9,11]^ described the randomized allocation schemes in detail, and 5 RCTs^[6,7,10,12,13]^ only described the use of randomization. Withdrawals and dropouts were reported in all trials, and 7 trials^[4–7,9,11,13]^ were not included in the final analysis on an intention-to-treat basis because of loss to follow-up or exclusion from the analysis of PEP (Table 4). All trials met at least 3 quality standards (randomization, blinding, withdrawals, and dropouts) and were of high quality (Jadad scores).

**Table 4:** Jadad quality scores of randomized controlled trials.

Author, year	Randomization	Exclusion criteria	Withdrawals and dropouts	Intention-to-treat analysis	Jadad score
Sudhindran et al 2001^[4]^	Computer generated	Clear	Clear	No	5
Kaffes et al 2006^[5]^	Computer generated	Clear	Clear	No	5
Moreto et al 2003^[6]^	Not clear	Clear	Clear	No	4
Beauchant et al 2008^[7]^	Not clear	No-clear	Clear	No	4
Nojgaard et al 2009^[8]^	Computer generated	Clear	Clear	Yes	5
Bhatia et al 2009^[9]^	Computer generated	Clear	Clear	No	5
Hao et al 2009^[10]^	Not clear	Clear	Clear	Yes	4
Tomoda et al 2019^[11]^	Computer generated	Clear	Clear	No	4
Sotoudehmanesh et al 2014^[12]^	Not clear	No-clear	Clear	Yes	4
Bo Luo & Wei 2016^[13]^	Not clear	Clear	No-clear	No	3

### 3.3. PEP incidence analysis

**Figure 1.**
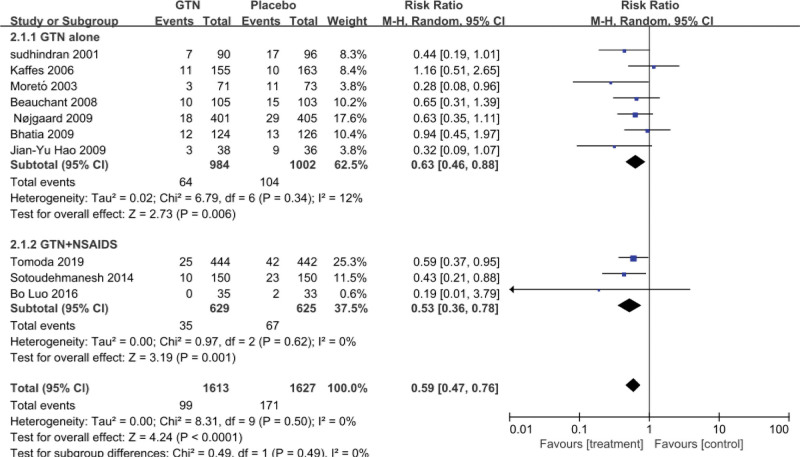
Meta-analysis of glyceryl trinitrate’s prophylactic effect on post-ERCP pancreatitis. The forest plot demonstrates a significant decrease in the overall incidence of PEP with prophylactic GTN use, either with GTN alone or combination with NSAIDS. CI = confidence interval, ERCP = endoscopic retrograde cholangiopancreatography, GTN = glyceryl trinitrate, M-H = Mantel-Haenszel, NSAIDs = nonsteroidal anti-inflammatory drugs, PEP = post-ERCP pancreatitis.

Additionally, 10 studies reported PEP, with 8.33% (270/3240) of the patients developing PEP, 6.14% (99/1613) in the GTN group, and 10.51% (171/1627) in the placebo group. There was no heterogeneity among these studies (*P* = .72, *I*^2^ = 0%), hence we used the fixed-effects model and discovered that the administration of GTN was associated with a significant reduction in the overall PEP incidence (RR = 0.59, 95% CI: 0.47–0.76; *P* < .0001); the PEP incidence was significantly lower in the treatment group than in the placebo group. Three studies^[11–13]^ treated groups with GTN combined with NSAIDs and the control group with NSAIDs alone. The results of the meta-analysis of 3 RCTs indicated that there was low heterogeneity among the trials (*P* = .62, *I*² = 0%); therefore, we used the fixed-effects model to reveal the incidence rate of the experimental group: 5.40% (54/1001), and that of the control group: 5.56% (35/629). The difference in incidence between the 2 groups was statistically significant (RR = 0.53, 95% CI: 0.36–0.78; *P* = .001), showing that PEP incidence was significantly lower in the GTN combined with NSAIDs group than in the NSAIDs alone group (Fig. 1).

### 3.4. Non-mild PEP incidence analysis

**Figure 2.**
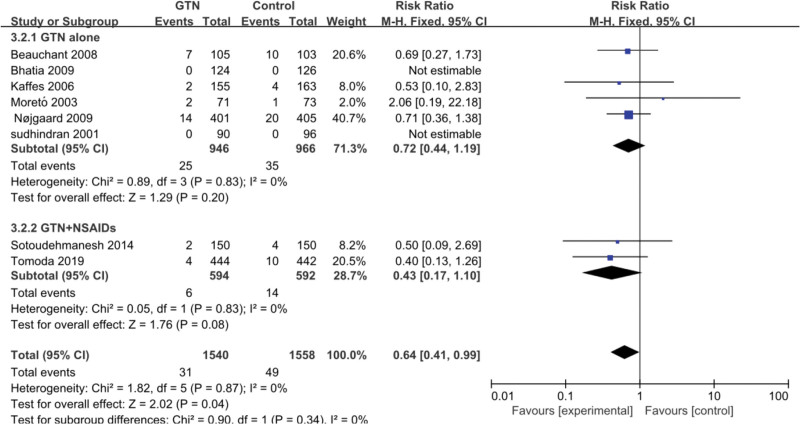
Meta-analyses between GTN and incidence of non-mild PEP. The forest plot demonstrates non-mild PEP incidence was significantly lower in the GTN combined with NSAIDS group than in the NSAIDS alone group. CI = confidence interval, GTN = glyceryl trinitrate, M-H = Mantel-Haenszel, NO-mild PEP = pancreatitis other than mild acute pancreatitis, NSAIDs = nonsteroidal anti-inflammatory drugs, PEP = post-ERCP pancreatitis.

We grouped 10 RCTs, of which 2 studies^[10,13]^ did not record the severity of PEP and were excluded, and 8 studies^[4–9,11,12]^ were divided into 2 subgroups: GTN alone versus placebo and GTN + NSAIDs versus NSAIDs. The results reported that 2.47% (80/3240) patients developed non-mild PEP, where 1.01% (6/594) were in the GTN with NSAIDs group and 2.36% (14/592) in the placebo group. There was no heterogeneity among these studies (*P* = .34, *I*² = 0%). Therefore, we used the fixed-effects model and discovered that the administration of GTN alone may not affect the reduction in PEP severity (RR = 0.72, 95% CI: 0.44–1.19; *P* = .2; Fig. 2). The results of the 2 RCTs^[11,12]^ indicated low heterogeneity among these trials (*P* = .83, *I*² = 0%); therefore, we used the fixed-effects model and discovered that the incidence rate of moderate-to-severe pancreatitis in the experimental group was1.00% (6/594), and the incidence rate in the control group was 2.36% (14/592). The difference in incidence between the 2 groups was statistically significant (RR = 0.43, 95% CI: 0.17–1.10; *P* = .08), showing that the incidence of non-mild PEP was not significantly lower in the GTN combined with NSAIDs group than in the NSAIDs alone group.

### 3.5. Drug side effects

**Figure 3.**
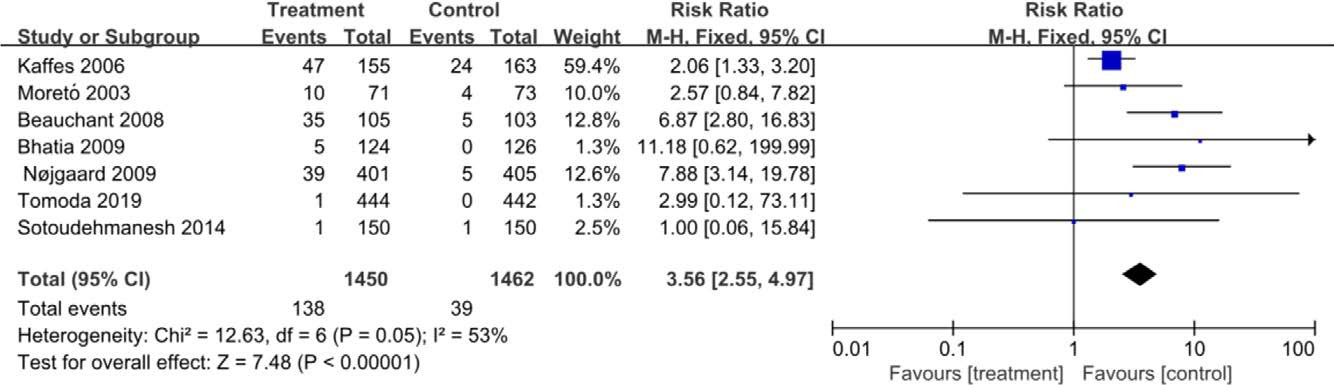
Meta-analyses between GTN and side effects of the drug. The forest plot demonstrates a significant increase in the risk of headache occurrence with GTN use. CI = confidence interval, GTN = glyceryl trinitrate, M-H = Mantel-Haenszel.

Among the included studies, headache was the most common adverse effect associated with GTN use. Six studies^[5–7,9,11,12]^ reported the occurrence of headaches: 9.52% (138/1450) in the GTN group and 2.67% (39/1462) in the placebo group. There was large heterogeneity among these studies (*P* = .05, *I*² = 53%); therefore, we used the random-effects model and observed that the administration of GTN was associated with a significantly increased risk of occurrence of headache in the GTN group than in the placebo group (RR = 3.79, 95% CI: 1.98–7.28; *P* < .0001; Fig. 3).

### 3.6. Publication bias

**Figure 4.**
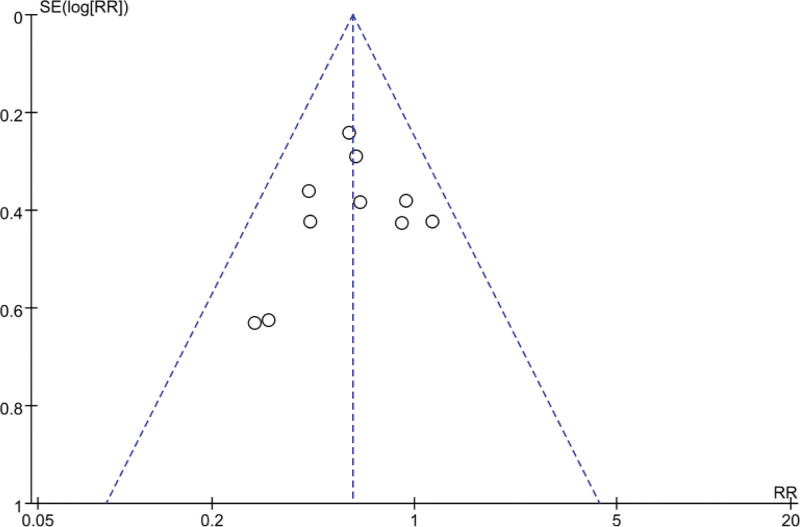
Funnel plot for publication bias in RR analysis. Each dot represents the RRs for the percentage of the incidence of PEP with prophylactic GTN use or placebo use. The dashed line represents the 95% CI line. GTN = glyceryl trinitrate, PEP = post-ERCP pancreatitis, RR = risk ratio, SE = Standard Error.

The funnel plot shows that the dots are distributed on both sides of the dotted line, indicating that there were no potential publication biases in the included trials (Fig. 4).

## 4. Conclusions

The most widely accepted mechanism by which ERCP occurs is that mechanical trauma to the papilla or pancreatic sphincter, especially Oddi’s sphincters, caused during instrumentation, creates transient obstruction of outflow of pancreatic juice. GTN can generate active NO− radical in the body and activate a series of biological signal transduction, thus play the role of relaxing smooth muscle. It also expands the sphincter of Oddi in ERCP patients to flow out panic juice and reduce the occurrence of PEP. Previously, meta-analysis has shown that prophylactic use of GTN can reduce the overall incidence of PEP, but there is also literature pointing out that pharmacologic prophylaxis alone is not adequate to prevent post-ERCP pancreatitis. In this regard, this analysis expanded the sample size and increased the RCTs analysis to show that GTN has a significant benefit in the prevention of the occurrence of PEP (*P* < .0001), However, it cannot be ruled out that the results are caused by low-risk patients. In high-risk patients who are very likely to develop moderate-to-severe PEP, it is still a question. In this meta-analysis, the use of GTN alone was not effective in preventing the occurrence of non-mild PEP (RR = 0.72, 95% CI: 0.44–1.19; *P* = .2). If GTN is used in combination with NSAIDS, it may have no effect on reducing non-mild PEP. The results are still questionable. First, because only 2 relatively small trials were included, the conclusions of the meta-analysis may not be strong enough. Second, these drug prevention studies were conducted in patients with low to average risk or in those patients who received pancreatic stents, not just drug interventions alone. There is a literature that indicates that placement of a small-bore–protective pancreatic stent after ERCP will reduce morbidity in 60% to 80% of high-risk and low-mixed-risk PEP patients.^[15]^ Attempting to prevent PEP by pharmacological intervention alone without the use of a protective pancreatic stent may be similar to trying to prevent cholangitis with antibiotics alone without catheter drainage—it may be effective in milder cases, but is unlikely to be effective in severe cases.^[15]^ In this regard, we recommend further collection of more RCTs: first, multicenter or large single-center studies, with more details and patient-related records of PEP and other complications. Second, it is best to include patients at high risk of PEP, and to stratify patients after ERCP—high-risk PEP versus low-risk PEP—further well-designed RCTs are needed to confirm the role of GTN in preventing intermediate-high-risk PEP.

## Author contributions

**Data curation:** Xuan Zhang, Jing-Ming Zhang, Wan Wei.

**Methodology:** Xuan Zhang.

**Software:** Xuan Zhang, Jing-Ming Zhang, Wan Wei, Hui Lin.

**Writing—original draft:** Xuan Zhang, Jing-Ming Zhang.

**Supervision:** Hui Lin.

**Writing—review & editing:** Hui Lin.
